# Through-plane dark-rim artefacts in 3D first-pass myocardial perfusion

**DOI:** 10.1186/1532-429X-17-S1-P100

**Published:** 2015-02-03

**Authors:** Merlin Fair, Peter Gatehouse, David Firmin

**Affiliations:** NHLI, Imperial College London, London, UK; Cardiovascular BRU, Royal Brompton Hospital, London, UK

## Background

The dark-rim artefact (DRA) is well known in 2D first-pass perfusion (FPP). The in-plane features of DRA are understood, but DRA has not been examined along the second phase-encoding (partition) direction for 3D FPP. The Gibbs contribution to DRAs in 2D FPP is minimised by finer resolution, but low through-plane resolutions of 3D FPP imply risk of partition axis DRAs. We investigated these new partition DRAs ("PDRAs") and partial volume effects due to coarse resolution of this direction.

## Methods

Low-resolution data at typical 3D FPP parameters were subsampled from 3 high-resolution sources to study PDRAs. High in-plane resolution was maintained so that any changes arose only from through-plane effects. The subsampled number of partitions used in the reconstructions (N_p_) was varied to give a 2-32mm range of through-plane resolutions.

1. A numerical phantom modelled a conical LV at intensity ratio 5:2 (blood:myocardium at first pass peak). N_p,_ and the angle between endocardial wall and image plane, θ_B_, were varied while plotting width of PDRAs.
2. In-vivo investigation: single-frame high-resolution data (1.3x1.3x2.0mm) was acquired by navigator-gated bSSFP at high flip-angle for similar blood:myocardium intensity ratio (approx 2:1).
3. LV blood and myocardium were manually segmented and the intensity of each tissue uniformly set to the 5:2 ratio of the numerical phantom. This gave more anatomically realistic data than the uniform cone, with changing θ_B_ along and around the LV.

## Results

**Figure 1:**
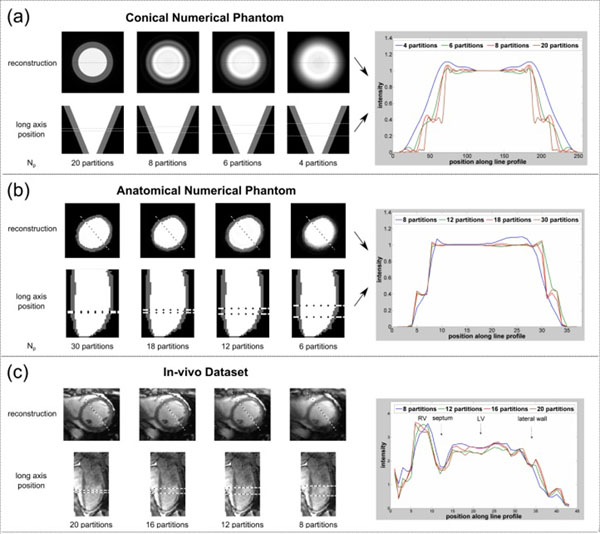
**Short-axis reconstructions of ‘slices' all at a fixed in-plane resolution but of varying through-plane resolutions, shown with their corresponding locations along the high-resolution long-axis (which was along the line marked on the SAX images).** To their right are the corresponding line profiles of each reconstruction at this position, allowing both the partition thickness and boundary angle responsible for producing any PDRA to be viewed simultaneously. These are shown for a) the conical numerical phantom, b) the anatomical numerical phantom and c) the in-vivo dataset. Values of N_p_ were chosen to give reconstructions representative of small PDRA, stonger PDRA, partial-volume/PDRA compromise and strong partial-volume effects respectively from left to right. The actual numbers therefore vary depending on the type of dataset used, although the first (high N_p_) high-resolution case is consistently unfeasibly high.

**Figure 2:**
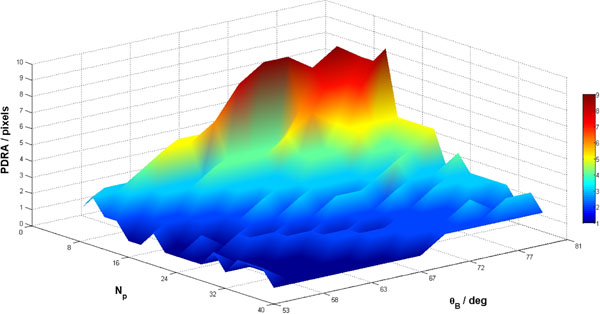
**Values of the partition-DRA (PDRA) width (in number of pixels) in the conical numerical phantom, reconstructed at varying boundary angles (θ**_**B**_ **) and number of secondary phase-encode steps (partitions) (N**_**p**_ **).** The PDRA width was determined through automatic detection of negative intensity deviation from the programmed myocardial value at the subendocardial layer. For the angles investigated, reconstructions with N_p_ < 8 were not scored, as partial volume effects became sufficiently strong to obscure the myocardium (see Figure 1). Excluding these cases, the PDRA widths increased with both reduced N_p_ (corresponding to coarser through-plane resolution) and increased θ_B_.

Slice reconstructions (Fig 1) show overlapping consequences of increased PDRAs and partial volume (blurring) as slice thickness is increased. Arrows (1a) show PDRA in the 8-partition image coming through-plane, n.b. not an in-plane DRA. Variation of θ_B_ and N_p_ in the conical phantom significantly altered PDRA width (Fig 2), with strongest artefacts at combined low resolution and sharp θ_B_. Although the border is sharpest at θ_B_ near 90°, this implies no variation between planes and therefore no PDRA. Whilst PDRA width increases at coarser resolution, the simultaneous impact of partial volume at lower N_p_ counteracts this effect. Eventually the blurring dominates and destroys endocardial border visibility. This pattern was seen in conical and anatomical phantoms (Fig 1a&b) and to some extent in-vivo (Fig 1c), although complicated by intensity slopes and effects beyond the LV. For some values of θ_B_ examined (~>65°) that may well occur in-vivo, sufficiently high through-plane resolution to avoid PDRAs is infeasible in 3D FPP; however, some compromise between PDRA and partial volume may be possible.

## Conclusions

Contrary to expectation that increased resolution reduces DRAs, at the low through-plane resolutions of 3D FPP increasing the resolution in this direction may increase Gibbs-induced DRAs due to sharper through-plane boundaries. However, this is a trade-off against partial volume blurring at low partition resolution. Further in-vivo investigations should optimise compromise between these two effects.

## Funding

Supported by the NIHR Cardiovascular Biomedical Research Unit of Royal Brompton and Harefield NHS Foundation Trust and Imperial College London, UK.

MF is funded by a British Heart Foundation (BHF) PhD Studentship Grant - FS/13/21/30143.

